# Case of Wound of the Stomach

**Published:** 1851-03

**Authors:** Robert K. Smith

**Affiliations:** Darby, Pa.


*Case of Wound of the Stomach.* By Robert K. Smith, M. D. of Darby, Pa.

A singular case has recently fallen under my observation, which seems show, that the stomach may receive a penetrating wound of considerable extent, without symptoms of organic disturbance, and without any escape of its contents into the abdominal cavity.

The case referred to, was that of a negro man, who was stabbed with a dull butcher knife, on the morning of the 12th inst.

I saw the case about half an hour afterwards, and found the wound on the left side, between the seventh and eighth ribs, behind the cartilages, extending backwards nearly two inches, with about four inches of the colon and a large portion of omentum protruding externally.

The omentum was a good deal lacerated; and the intestine fully distended with gas, and so much strangulated at the wound, as to render its enlargement necessary, in order to effect a reduction.

This was done sufficiently to allow the protruded viscera to be replaced within the cavity of the thorax, and to be pushed down with the finger, through the wound in the diaphragm, into the abdomen. I then closed the wound with interrupted sutures, and placed a compress upon it, secured by a bandage around the body.

The pulse was 78 and feeble. Great prostration existed, and, from the examination, I felt confident that there was profuse internal hemorrhage.

The patient complained of thirst, great pain in the stomach, dyspnœa and faintness ; but, from the moment the wound was inflicted, until his death, there was neither nausea nor vomiting.

I saw him twice during the succeeding six hours, and found the change in his symptoms to be, great restlesness, increase of pain, and difficulty of breathing, and a gradual sinking of the vital powers from the hemorrhage ; a large amount of which had escaped through the interstices of the wound, and become infiltrated around it. The following morning the pulse was almost imperceptible ; no pain ; increased dyspnœa ; and gradual sinking. He died at noon ; twenty-six hours after he was stabbed.

*Autopsy.*—Upon dissecting up the integuments, a portion of omentum was discovered to have returned through the diaphragm, plugging the wound between the ribs.

Parts of the 5th, 6th, and 7th ribs, were next removed, and a large opening appeared in the diaphragm, filled with omentum. There were probably between two and three pounds of fluid in the thorax, with complete collapse of the lung.

The abdomen was next opened, and the knife was found to have passed downwards, through the diaphragm, into the stomach, through the anterior or superior wall at its cardiac extremity, making an opening, in the organ, of more than an inch in length.

The stomach was full, and around the wound in it there had been some inflammatory action, but not the smallest portion of its contents had escaped into the abdominal cavity.

The peculiarities which this case presents, are the entire quietude of the stomach after sustaining such extensive injury, and the retention of the whole of its contents when so fully distended.

The stab was inflicted shortly after the man had eaten his breakfast, and he drank freely of water and other liquids, up to the time of his death.

The only way in which I can account for the retention, is, that the wound must have been closed, by a part of the returned omentum falling upon it,

*Darby, Pa.*, 28*th Jan.*, 1851.

